# Breaking the silence: confidence and barriers in raising concerns among undergraduate dental students– “a national study”

**DOI:** 10.1186/s12909-025-07092-z

**Published:** 2025-04-21

**Authors:** Layla Hassouneh, Kamran Ali, Rebecca Glanville, Hani Nazzal, Sanaa Aljamani, Nabilah Quadier, Manal Matoug-Elwerfelli

**Affiliations:** 1 Department of Conservative Dentistry, Jordan University of Science and Technology, Irbid, Jordan; 2 College of Dental Medicine, QU Health, Qatar University, Doha, Qatar; 3 Faculty of Health, Peninsula Medical School, University of Plymouth, Plymouth, UK; 4 Dentistry Department, Hamad Dental Centre, Hamad Medical Corporation, Doha, Qatar; 5 Restorative department, School of Dentistry, University of Jordan, Amman, Jordan; 6 Faculty of Dentistry, University of Liverpool, Liverpool, UK; 7 Jordan University of Science and Technology, Irbid, Jordan

**Keywords:** Dental students, Curriculum, Education, Patient safety, Raising concerns, Whistleblowing

## Abstract

**Background:**

Raising concerns in clinical settings, also known as whistleblowing, is vital for safeguarding patient safety and improving the quality of care. Despite research on whistleblowing in medical and nursing fields, there is limited evidence on this topic within dental education. This study aims to assess the self-reported confidence of undergraduate dental students in raising concerns and identify any barriers.

**Methods:**

This cross-sectional study utilized an online close-ended questionnaire distributed via Google Forms to senior undergraduate dental students from Jordan University of Science and Technology and the University of Jordan, Jordan. Data collection was voluntary, with subsequent analysis performed using RStudio (version 2023.06.2) incorporating R version 4.0.5. T-tests and Analysis of Variance (ANOVA) were used to assess significant variations between results by gender and stage of study.

**Results:**

A total of 382 participants were included in the study yielding a response rate of 30.80%. Of these, 257 were female (67.28%) and 125 were male (32.72%). Overall, 169 (44.24%) participants reported that their institutions had a policy document on raising concerns, while only 71 (18.58%) participants reported receiving formal training in raising concerns at their institution. Approximately 45% of participants reported experiencing situations which warranted raising concerns in clinical settings. The overall mean score for all items was 0.13 (95% CI -0.18 to 0.43). The findings revealed that students were marginally confident in raising concerns related to patient safety. However, their confidence was lower when addressing issues related to the conduct of clinical staff or peers. Common barriers reported included fear of causing trouble, lack of support, and fear of being ignored. ANOVA revealed significant variation by gender and year of study, with female students and final-year students reporting greater self-confidence in raising concerns (*p* < 0.001).

**Conclusions:**

Notwithstanding the limitations of the current study, the results show that participants were marginally confident in raising concerns related to patient safety and several barriers to raising concerns were also identified. These findings underscore the need for dental schools to focus on enhancing students’ confidence and empowering them to report concerns when warranted. A transparent and supportive culture can contribute to improvements in patient safety and enhancing professionalism of dental students.

**Supplementary Information:**

The online version contains supplementary material available at 10.1186/s12909-025-07092-z.

## Introduction

Raising concerns, also known as whistleblowing, in clinical settings involves “speaking up” or reporting by healthcare professionals when they identify risky or inadequate actions of others within the healthcare team, with the ultimate aim of improving patient safety and quality of clinical care [1]. Over the last decade, the emphasis on whistleblowing has increased, particularly following the publication of the Francis Report in 2013, which investigated the failings at the Mid-Staffordshire National Health Service (NHS) Foundation Trust [2]. This event prompted changes within the United Kingdom (UK) and led to a growing body of publications emphasizing the need for healthcare professionals and trainees to raise concerns about behaviors that could compromise patient safety, colleague well-being, or their own safety [3–6].

Recognizing the importance of raising concerns, it is now seen not only as an ethical and professional responsibility but also regarded as a legal requirement [7]. Consequently, integration of ethical practices, including a focus on raising concerns, into undergraduate dental curricula is vital [8, 9]. Many medical and dental schools, particularly in the UK and the USA, have implemented policies to support and protect students throughout this process [10–12]. Such measures are crucial during clinical rotations, where students are directly involved in patient care. A culture of openness and honesty is foundational to the success of any healthcare organization [13].

All healthcare professionals, regardless of their level of training, have a duty of care to safeguard patient safety and uphold ethical standards [8]. In clinical training environments, where dental students perform invasive and irreversible procedures under supervision, ethical dilemmas can be especially prevalent [14]. While studies have focused on medical and nursing students’ attitudes toward raising concerns [15–17], data specific to dental students remain scarce. Previous studies indicate that healthcare students often lack confidence to act on ethical dilemmas involving peers or clinical staff [14, 18]. Although many students recognize the importance of reporting concerns, they frequently face significant barriers [6, 15]. These barriers include personal fear, potential disciplinary actions, economic and emotional deprivation, victimization, and negative feedback, all of which contribute to a culture of silence by deterring individuals from speaking up [15, 19, 20]. Moreover, simply raising awareness about the importance of reporting is insufficient; addressing these deterrents is essential to ensure that all parties are protected and to foster an environment in which concerns can be raised without fear of retribution [7, 15].

Given the central role of clinical training in dental education, it is imperative for dental schools to create environments that empower students to raise concerns confidently, without fear of retaliation [8]. Despite active encouragement for whistleblowing as a means of protecting patients, colleagues, and the public, there remains a notable gap in research specifically examining the attitudes and behaviors of undergraduate dental students regarding ethical dilemmas in clinical practice. The current study aims to assess the self-reported confidence in raising concerns within clinical dental settings and identify barriers faced by undergraduate dental students.

## Materials and methods

### Research ethics

This research was conducted in accordance with the Declaration of Helsinki ethical principles for medical research involving human subjects, including research on identifiable human material and data. Ethical approval was obtained from the Institutional Review Board Committees at Jordan University of Science and Technology, Jordan (Approval number: 160/2023) and University of Jordan, Jordan (Approval number: 235/2024). Participation in the study was voluntary, and all data were recorded and processed anonymously. All participants provided informed consent prior to data collection.

### Study design

It was a cross-sectional study based on an online survey.

### Sampling technique and participants

A non-probability purposive sampling technique was used to recruit undergraduate dental students at dental schools based at Jordan University of Science and Technology (JUST) and University of Jordan, Jordan. Students in Years 4, 5 and 6 were eligible to participate in the study. Invites to participate in the study were sent to the students’ university email addresses accompanied by a participant information sheet providing the aims and scope of the study.

### Data collection instrument

The study questionnaire was adapted from questionnaire used previously on medical students [14]. The questionnaire consisted of the following sections:

Section 1 Demographics: Four Items.

Section 2: Institutional policy and previous experience of raising concerns: Five items.

Section 3: Self-confidence in raising concerns: Six items with five subsections in each item.

Section 4: Barriers and institutional support to raising concerns: Two items.

Sections 3 and 4 were similar to the items used in questionnaire on medical students [14]. The research team added a section related to demographics to include participants’ gender, stage of study, if they had started their clinical training, and university. Additionally, five items were included in Sect. 2 to evaluate the existence and access to institutional policy on raising concerns, training of students, and previous experiences of the participants in encountering situations which warranted raising concerns. Conditional branching, also known as question-skip logic, was utilized for specific questions to create a custom path based on respondents’ answers.

Pretesting of the questionnaire was done with ten academics and twenty students. The purposes of pretesting of scale items were as follows:

1. Determine the content and face validity of the items.
2. Determine the participants’ clarity and consistent interpretation of the questionnaire.
3. Determine the correlations between ordinal variables.

Feedback from the participants in the pretesting phase identified a need for minor language improvements for two items before finalizing the questionnaire. Kendall’s Tau showed satisfactory correlations between ordinal variables (τ = 0.78). The final version of the questionnaire is included in the appendix.

### Data collection

A total of 1240 eligible students were invited to participate in the study. Participation was voluntarily and Informed consent was obtained through Google Forms before participants completed the questionnaire. The online Google Forms questionnaire was distributed in February 2024, followed by three reminder emails sent every three weeks from the initial invitation.

### Data analysis

All data were analyzed and visualized using RStudio (version 2023.06.2) with R version 4.0.5 (R Core Team 2022). Percentages were calculated for nominal and categorical variables. Descriptive statistics, including confidence intervals, were calculated for ordinal variables. Responses to items on self-confidence in raising concerns (Sect. 3) were scored as follows: Least confident = minus 2; Not confident = minus 1; Neutral = 0; Fairly confident = 1; Very confident = 2.

T-tests and Analysis of Variance (ANOVA) were used to assess significant variations between results by gender and stage of study. Estimated marginal means were derived from the ANOVA outcomes.

## Results

### Section 1: Demographics and response rate

A total of 382 participants were included in the study yielding a response rate of 30.80%. Of these, 257 were female (67.28%) and 125 were male (32.72%). All participants were undergraduate dental students, with 325 students (85.08%) enrolled in Year 4, 44 students (11.52%) in Year 5 and 13 students (3.40%) in Year 6/internship. Participants were from one of two universities: 298 students (78.01%) were enrolled at Jordan University of Science and Technology, while 84 students (22.00%) were from the University of Jordan. All participants confirmed that they were doing their clinical training. A post-hoc power analysis using Cohen’s f effect size indicated that the study sample size had sufficient power (> 0.99) to detect differences between year groups at α = 0.05.

### Section 2: Institutional policies and previous experience

A total of 169 (44.24%) participants reported that their institutional had a policy document on raising concerns; 44 participants (11.51%) reported in the negative; and 169 (44.24%) participants were unsure if an institutional policy document existed. Of the 169 participants who reported an existing institutional policy on raising concerns, 108 participants (63.90%) regarded the policy document as easily accessible to students. Only 71 (18.58%) participants reported receiving formal training in raising concerns at their institution. Of the 311 participants who did not receive institutional training on raising concerns, 50.48% affirmed their interest in receiving training on raising concerns.

When asked about previously encountered situation that warranted raising concern, the majority of the participants (45%) reported yes (previously encountered history). In contrast, 26% and 29% of participants responded “no” and “not sure”, respectively.

### Section 3: Self-confidence in raising concerns

The overall mean score for all items was 0.13 (95% CI -0.18 to 0.43). Descriptive statistics for each individual item are presented in Table 1. The overall rating was slightly positive, suggesting that, on the whole, students were marginally confident in raising concerns. Students indicated they would be most confident raising issues concerning patient safety, such as poor infection control, and were generally more confident addressing issues with fellow students.

**Table 1:** Descriptive statistics for self-reported confidence in Raising concerns

Question	Mean	SD±	95% CI (lower)	95% CI (upper)
3.1 *How confident would you feel reporting an issue concerning patient safety*,* such as poor infection control?*	0.5	1.3	0.37	0.63
Rate your confidence in reporting this issue to each of the following:				
a. Clinical supervisor	0.34	1.24	0.22	0.46
b. Dental nurse/ assistant	0.23	1.2	0.11	0.35
c. Academic mentor/ course coordinator	0.17	1.19	0.05	0.29
d. Fellow student	0.39	1.25	0.26	0.51
e. Clinical manager (Head of clinics)	0.19	1.29	0.06	0.32
3.2 *How confident would you feel reporting an issue of probity (honesty) regarding patients’ clinical records*,* such as*,* changing*,* falsifying or misrepresenting information in patient’s notes?*	0.22	1.22	0.1	0.34
Rate your confidence in reporting this issue to each of the following:				
a. Clinical supervisor	0.13	1.19	0.01	0.24
b. Dental nurse/ assistant	0.05	1.14	-0.06	0.17
c. Academic mentor/ course coordinator	0.1	1.17	-0.02	0.21
d. Fellow student	0.23	1.17	0.11	0.35
e. Clinical manager (Head of clinics)	0.05	1.18	-0.07	0.17
3.3 *How confident would you feel reporting an issue regarding unprofessional behavior (attitude and conduct of trust) towards a patient*,* such as rudeness*,* disrespect or bullying?*	0.26	1.29	0.13	0.39
Rate your confidence in reporting this issue to each of the following:				
a. Clinical supervisor	0.27	1.31	0.13	0.4
b. Dental nurse/ assistant	0.11	1.24	-0.02	0.23
c. Academic mentor/ course coordinator	0.16	1.27	0.03	0.28
d. Fellow student	0.26	1.26	0.14	0.39
e. Clinical manager (Head of clinics)	0.18	1.29	0.05	0.31
3.4 How confident would you feel reporting an issue regarding attitude and conduct between clinical staff, for example, an argument?	-0.02	1.25	-0.14	0.11
Rate your confidence in reporting this issue to each of the following:				
a. Clinical supervisor	-0.04	1.24	-0.16	0.09
b. Dental nurse/ assistant	-0.11	1.18	-0.23	0.01
c. Academic mentor/ course coordinator	-0.02	1.2	-0.14	0.11
d. Fellow student	0.13	1.2	0.01	0.25
e. Clinical manager (Head of clinics)	-0.02	1.24	-0.14	0.11
3.5. *How confident would you feel reporting an issue regarding attitude and conduct of clinical staff toward a student*,* such as disrespect?*	0.21	1.33	0.07	0.34
Rate your confidence in reporting this issue to each of the following:				
a. Clinical supervisor	0.12	1.34	-0.01	0.26
b. Dental nurse/ assistant	-0.05	1.24	-0.18	0.07
c. Academic mentor/ course coordinator	0.14	1.34	0.01	0.28
d. Fellow student	0.26	1.27	0.14	0.39
e. Clinical manager (Head of clinics)	0.13	1.34	-0.01	0.26
3.6 *How confident would you feel reporting an issue regarding attitude and conduct between fellow students*,* such as rudeness*,* disrespect*,* or bullying?*	0	1.25	-0.13	0.13
Rate your confidence in reporting this issue to each of the following:				
a. Clinical supervisor	-0.02	1.29	-0.15	0.11
b. Dental nurse/ assistant	-0.07	1.23	-0.19	0.06
c. Academic mentor/ course coordinator	-0.04	1.24	-0.16	0.09
d. Fellow student	0.14	1.26	0.01	0.26
e. Clinical manager (Head of clinics)	-0.02	1.25	-0.14	0.11
All	0.13	1.26	-0.18	0.43

SD±; Standard deviation, CI; Confidence interval

Descriptive statistics for each individual item, categorized by gender and stage (year of study), are listed in Tables 2 and 3. Analysis of Variance (ANOVA) of the mean scores across all items revealed significant variation by gender and year of study, with female students and final-year students reporting greater self-confidence in raising concerns (*p* < 0.001), a series of T-tests reported no significant variation by gender when considering individual items. ANOVA of individual items by stage only identified significant variation in item 3.1d (How confident are you reporting an issue regarding “patient safety such as poor infection control” to a fellow student) (*p* = 0.001) and item 3.5b (How confident are you reporting an issue regarding “attitude and conduct between clinical staff toward a student, such as disrespect” to a dental nurse/assistant) (*p* < 0.001). Post-hoc Tukey’s HSD testing found significant variation between Year 4 and Year 5 in both items with the Year 5 students having a higher mean score (diff = 0.69, *p* = 0.002) in item 3.1d and item 3.5b (diff = 0.83, *p* < 0.001).

**Table 2:** Descriptive statistics for self-reported confidence in Raising concerns by gender

	Mean		SD±		95% CI (lower)		95% CI (upper)	
Question ID	Female	Male	Female	Male	Female	Male	Female	Male
Q 3.1	0.55	0.38	1.29	1.32	0.4	0.15	0.71	0.62
Q 3.2	0.2	0.27	1.18	1.31	0.05	0.04	0.34	0.5
Q 3.3	0.32	0.15	1.28	1.33	0.16	-0.08	0.47	0.39
Q 3.4	-0.03	0.01	1.26	1.25	-0.19	-0.21	0.12	0.23
Q 3.5	0.19	0.23	1.33	1.34	0.03	0	0.36	0.47
Q 3.6	-0.03	0.06	1.24	1.28	-0.18	-0.17	0.12	0.28
Overall	0.15	0.09	1.23	1.3	0.02	-0.04	0.27	0.22

SD±; Standard deviation, CI; Confidence interval

**Table 3:** Descriptive statistics for self-reported confidence in Raising concerns by stage

	Mean			SD±			95% CI (lower)			95% CI (upper)		
Question ID	Yr 4	Yr 5	Yr 6	Yr 4	Yr 5	Yr 6	Yr 4	Yr 5	Yr 6	Yr 4	Yr 5	Yr 6
Q 3.1	0.42	0.91	1	1.31	1.2	1.22	0.28	0.55	0.33	0.57	1.26	1.67
Q 3.2	0.17	0.41	0.85	1.23	1.17	1.07	0.04	0.06	0.26	0.31	0.76	1.43
Q 3.3	0.23	0.5	0.23	1.3	1.25	1.42	0.09	0.13	-0.55	0.38	0.87	1.01
Q 3.4	-0.03	0	0.23	1.25	1.29	1.36	-0.17	-0.38	-0.51	0.11	0.38	0.97
Q 3.5	0.18	0.34	0.46	1.33	1.31	1.45	0.03	-0.05	-0.33	0.32	0.73	1.25
Q 3.6	-0.03	0.16	0.31	1.25	1.24	1.49	-0.17	-0.21	-0.51	0.1	0.53	1.12
Overall	0.08	0.3	0.67	1.24	1.32	1.25	-0.04	0.17	0.55	0.21	0.44	0.8

SD±; Standard deviation, CI; Confidence interval

### Section 4: Barriers and institutional support

**Fig. 1:**
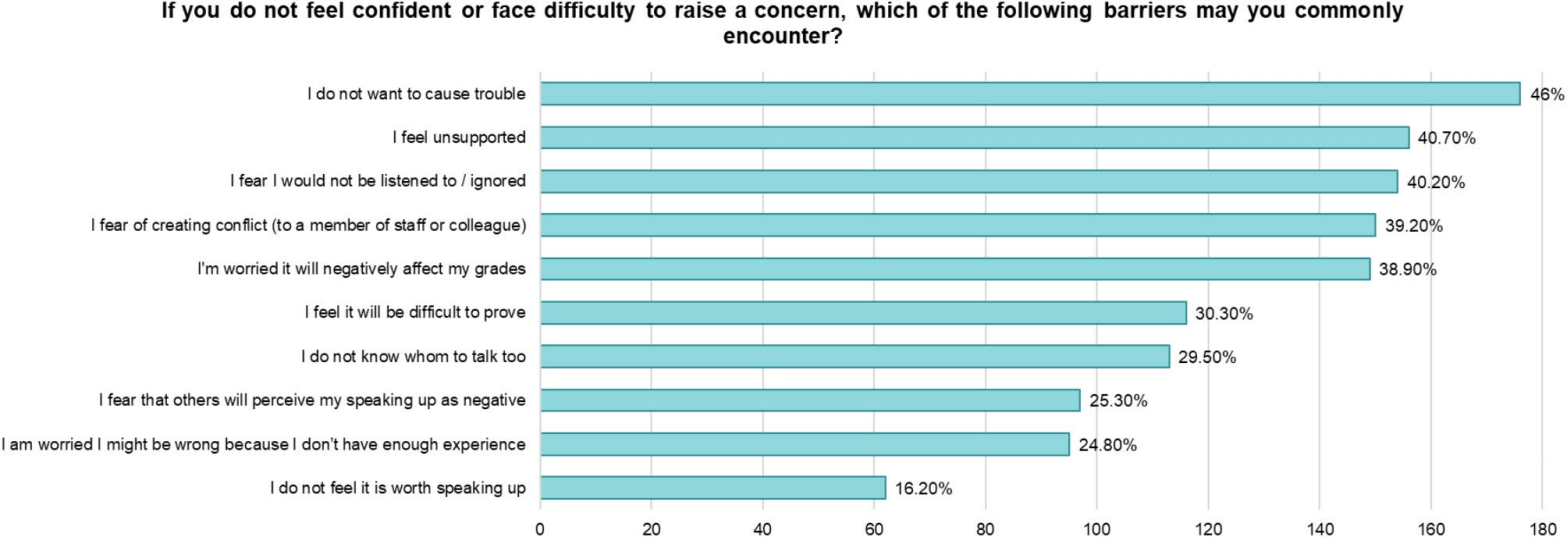
Bar graph representing common barriers encountered by undergraduate dental students

To better understand the lack of confidence or difficulty that undergraduate dental students face in raising concerns, several barriers were assessed and presented in Fig. 1. Participants reported a range of barriers, with the most cited being fear of causing trouble, feeling a lack of support, feeling ignored or not listened to, fear of causing conflict, and concern about negatively affecting their grades.

To enhance students’ confidence in raising concerns, participants suggested several institutional actions to provide support. In descending order of frequency, these include: (i) assuring safety for students who raise concerns (62.56%), (ii) ensuring that changes will be implemented following a report (56.54%), (iii) clarifying which areas must be reported (48.59%), (iv) identifying a designated person to whom concerns can be reported (47.64%), (v) introducing a clear channel for raising concerns (45.54%), and (vi) offering regular training and support (40.05%).

## Discussion

Dental students’ clinical awareness and confidence in raising clinical concern have not been sufficiently explored in the literature. Existing data on healthcare undergraduate students’ confidence and attitude towards raising concerns are scarce and primarily focused on medical and nursing students [14, 16, 17]. This study is among the few to investigate the level of self-reported confidence and the barriers to raising concerns from the perspective of undergraduate dental students during their clinical education.

The overall results of this study indicate that, undergraduate dental students are marginally confident in raising concerns, with self-reported confidence levels varying depending on the nature of the issue. The data revealed that students undergoing clinical training exhibit a higher level of self-reported confidence in addressing concerns related to patient safety. However, self-reported confidence levels were notably lower when it came to issues involving the attitude and conduct of clinical staff or fellow students. These findings align with a previous study conducted at Queen Mary University of London (UK), which also found that medical students were most confident when reporting patient safety issues [14]. In descending order, lower confidence levels were observed in reporting issues related to probity, staff and student conduct, the attitude of staff toward patients, and interactions between colleagues [14]. Additionally, a study involving medical students and surgical staff at a public hospital in South Africa found that just over half of the participants felt confident in reporting adverse events, while others expressed uncertainty or reluctance to report [16].

In the current study, participants’ confidence in raising concerns varied not only by context but also by the type of person students felt comfortable approaching. Higher self-reported confidence levels were noted when students raised concerns with fellow students or their direct clinical supervisor. In contrast, students were less confident when addressing concerns to other staff members, such as the clinical course director or the clinical manager. This pattern aligns with findings from a previous study, which showed that students were more confident discussing issues with peers or junior doctors but less so when engaging with formal authority figures [14]. This may be partly due to students often discussing their concerns with peers or junior staff first to gauge the validity and shared nature of their concerns before proceeding to formal reporting. A qualitative study exploring professionalism dilemmas among dental, nursing, pharmacy, and physiotherapy students identified common issues such as emotional mistreatment, patient safety breaches, and whistleblowing challenges [21]. The study also concluded that students from different disciplines express emotions differently and suggested that sharing these experiences and practicing ideal responses can strengthen their commitment to professional values [21].

Although in the current study, 45% of the participants reported observing inappropriate practices at least once during their clinical training, the data also revealed 29% of the participants were unsure whether they had encountered situations that warranted raising a concern. This uncertainty may point to a need for further support in helping students identify and evaluate what constitutes a legitimate concern. A recent systematic review concluded lack of ethical knowledge among healthcare students and professionals [22]. In clinical settings, this insufficient knowledge could make it challenging for students to differentiate between minor issues and more significant ethical concerns which warrant whistleblowing. Further research should focus on understanding the level of students’ knowledge and awareness regarding malpractice and ethical violations and how this affects their likelihood of raising concerns.

The current study also explored the barriers to raising concerns, with the three most frequently mentioned being fear of causing trouble, fear of being unsupported, and fear of being ignored. These findings align with a mixed-methods study conducted at University College London Medical School (UK), where students identified similar deterrents. The study reported that 74% believed nothing would be done, 63% feared damaging working relationships, 49% were concerned about causing problems for colleagues, 46% viewed incidents as isolated, 44% worried about potential career impact, and 41% did not know how to raise concerns [6]. The qualitative data from the above study further highlighted three key subthemes: (1) Comprehension; lack of understanding regarding what constitutes a valid concern, (2) Conviction; a diminished belief in the value of raising concerns due to past disappointments, (3) Courage; fear played a significant role in discouraging students from speaking up [6]. Indeed, students most often perceive the risks of raising concerns as disproportionately high, particularly when they believe that their actions will not result in meaningful change and may even expose them to potential punishment [19]. Another contributing factor to this reluctance is the perceived ineffectiveness of raising concerns primarily with fellow students or junior staff members, who may lack the necessary knowledge or authority to address the issue [14].

Addressing and overcoming barriers to raising concerns is essential for fostering a culture that empowers students to voice their concerns, ultimately enhancing patient safety [6]. Although a recent integrative review of 82 studies concluded that whistleblowing in clinical services can lead to positive outcomes, such as enhanced patient safety and favorable resolutions in employment disputes, legal settlements, and court rulings [23]. However, whistleblowers frequently suffer adverse repercussions and negative consequences, including occupational, legal, financial, and socioemotional challenges, despite the presence of protective policies in most healthcare organizations [23]. To address these issues, future whistleblowing policies should focus on minimizing the negative impact on whistleblowers while simultaneously improving patient safety and the quality of care provided [23]. Indeed, a culture of safety and trust is essential in fostering an environment where concerns can be reported without fear of retribution.

Considering the barriers highlighted above, it is essential for institutions to adopt a proactive and transparent approach in addressing concerns to ensure that students feel their opinions are valued and acted upon without fear of retribution. Establishing an ethically safe and supportive workplace culture is crucial. A study conducted on medical students at Wayne State University in the USA concluded that promoting error reporting in clinical settings is complex and involves more than just encouraging students; it requires fostering a culture of safety [5]. The study suggested that integrating patient safety principles into medical curricula, providing training on error recognition, and creating an environment where students feel safe to report incidents are crucial for improving reporting practices [5]. In the current study, self-reported confidence in raising concerns was found to be influenced by gender and year of study. Confidence increased with the seniority, correlating with increased clinical experience. However, this contrasts with findings from a single-center study in UK, which reported that increased clinical exposure was associated with a decline in students’ sense of responsibility to raise concerns [6]. Due to limited data from similar studies conducted on undergraduate students, making direct comparisons remains challenging.

### Limitations

The authors acknowledge some limitations of the current study that warrant careful consideration. A response rate of 30% was achieved. Given the sensitive nature of the topic and participants’ concerns regarding potential retaliation or punishment, it is possible that individuals who were even more apprehensive about raising concerns chose not to participate. This self-selection bias may have led to an over-optimistic representation of students’ confidence and attitudes. Although a post-hoc power analysis using Cohen’s f effect size confirmed that the sample size provided sufficient statistical power, the low response rate remains a limitation for the generalizability of the findings. Additionally, this study was conducted at the only two public dental schools in Jordan that offer clinical training for senior students. To date, other recently established dental schools in Jordan, which currently enroll only preclinical students, were excluded from this study. While this represents the first national-level attempt to explore this topic in Jordan, it also means that the findings cannot be readily generalized. This limitation restricts the scope of the findings of the current study to the specific context of these two institutions. Finally, cultural and environmental factors unique to Jordan may limit the applicability of these findings to dental education in other countries. Differences in educational policies, healthcare systems, and cultural attitudes toward whistleblowing and raising concerns could influence both the behavior and perceptions of dental students elsewhere. Despite these limitations, this study provides valuable insights into the barriers and facilitators of raising concerns in clinical dental settings, and it lays the groundwork for future research in more diverse educational and cultural contexts.

### Recommendations

Future studies at other institutions in different countries are recommended to address this. Additionally, incorporating data from official incident reporting data extracted from the healthcare systems and qualitative methods such as focus groups would provide a more comprehensive understanding of the barriers and facilitators related to raising concerns in undergraduate clinical dental education. The results of the current study aim to sensitize the stakeholders regarding ethical issues and safety of clinical environments in dental educational institutes. Dental educators must focus on teaching ethics and providing a supportive environment for the students to enable them to raise concerns without fear of retribution.

## Conclusion

Notwithstanding the limitations of the current study, the results show that participants were marginally confident in raising concerns related to patient safety and several barriers to raising concerns were also identified. These findings underscore the need for dental schools to focus on enhancing students’ confidence and empowering them to report concerns when warranted. A transparent and supportive culture can contribute to improvements in patient safety and enhancing professionalism of dental students.

## Electronic supplementary material

Below is the link to the electronic supplementary material.

Supplementary Material 1

## Data Availability

The data that supports the findings of this study are available on request from the corresponding author. The data are not publicly available due to ethical restrictions.
