# Supplementary material for: Breaking the silence: confidence and barriers in raising concerns among undergraduate dental students– “a national study”
Source: BMC Med Educ. 2025 Apr 21;25:584. doi: 10.1186/s12909-025-07092-z (PMC12013199; doi:10.1186/s12909-025-07092-z)
Supplement: Supplementary file 1 — Supplementary Material 1 [file 12909_2025_7092_MOESM1_ESM.pdf]

Appendix: Questionnaire on Raising Concerns by Undergraduate Dental Students in Clinical Settings

Section 1: Demographics

1.1 What is your gender?
☐ Male
☐ Female

1.2 What is your current stage of undergraduate dental education?
☐ Year 4
☐ Year 5
☐ Year 6

1.3 Have you started your clinical training?
☐ Yes
☐ No

1.4 At which institution are you studying?
☐ University of Science and Technology
☐ Jordan University

Section 2: Institutional policy and participant experience in raising concern

2. 1 Does your current institution have a raising concerns policy?
☐ Yes
☐ No
☐ Not aware

2.2 Is the institution raising concern policy easily accessible to all students?
☐ Yes
☐ No
☐ Not sure
☐ Not applicable

2.3 Have you received formal training on raising concerns at your institution?
☐ Yes
☐ No

2.4 If the answer to the previous question is no, would you like to receive formal training on raising concerns?
☐ Yes
☐ No

2.5 Have you previously encountered a situation which required you to raise concern?
☐ Yes
☐ No
☐ Not sure

Note: raising concerns refers to "speaking up" or reporting by healthcare professionals for the benefit of patient safety and quality of care upon recognizing or becoming aware of any unprofessional, unethical or deficient actions from individuals within the healthcare setting. This may include clinical, performance or personal misconduct.

Section 3: Self-confidence in raising concerns

3.1 *How confident would you feel reporting an issue concerning patient safety, such as poor infection control?*

☐ Least confident ☐ Not confident ☐ Neutral ☐ Fairly confident ☐ Very confident

Rate your confidence in reporting this issue to each of the following:

a. Clinical supervisor
☐ Least confident ☐ Not confident ☐ Neutral ☐ Fairly confident ☐ Very confident

b. Dental nurse/ assistant
☐ Least confident ☐ Not confident ☐ Neutral ☐ Fairly confident ☐ Very confident

c. Academic mentor/ course coordinator
☐ Least confident ☐ Not confident ☐ Neutral ☐ Fairly confident ☐ Very confident

d. Fellow student
☐ Least confident ☐ Not confident ☐ Neutral ☐ Fairly confident ☐ Very confident

e. Clinical manager (Head of clinics)
☐ Least confident ☐ Not confident ☐ Neutral ☐ Fairly confident ☐ Very confident

3.2 *How confident would you feel reporting an issue of probity (honesty) regarding patients' clinical records, such as changing, falsifying or misrepresenting information in patient's notes?*

☐ Least confident ☐ Not confident ☐ Neutral ☐ Fairly confident ☐ Very confident

Rate your confidence in reporting this issue to each of the following:

a. Clinical supervisor
☐ Least confident ☐ Not confident ☐ Neutral ☐ Fairly confident ☐ Very confident

b. Dental nurse/ assistant
☐ Least confident ☐ Not confident ☐ Neutral ☐ Fairly confident ☐ Very confident

c. Academic mentor/ course coordinator
☐ Least confident ☐ Not confident ☐ Neutral ☐ Fairly confident ☐ Very confident

d. Fellow student
☐ Least confident ☐ Not confident ☐ Neutral ☐ Fairly confident ☐ Very confident

e. Clinical manager (Head of clinics)
☐ Least confident ☐ Not confident ☐ Neutral ☐ Fairly confident ☐ Very confident

3.3 *How confident would you feel reporting an issue regarding unprofessional behavior (attitude and conduct of trust) towards a patient, such as rudeness, disrespect or bullying?*

☐ Least confident ☐ Not confident ☐ Neutral ☐ Fairly confident ☐ Very confident

Rate your confidence in reporting this issue to each of the following:

a. Clinical supervisor
☐ Least confident ☐ Not confident ☐ Neutral ☐ Fairly confident ☐ Very confident

b. Dental nurse/ assistant
☐ Least confident ☐ Not confident ☐ Neutral ☐ Fairly confident ☐ Very confident

c. Academic mentor/ course coordinator
☐ Least confident ☐ Not confident ☐ Neutral ☐ Fairly confident ☐ Very confident

d. Fellow student
☐ Least confident ☐ Not confident ☐ Neutral ☐ Fairly confident ☐ Very confident

e. Clinical manager (Head of clinics)
☐ Least confident ☐ Not confident ☐ Neutral ☐ Fairly confident ☐ Very confident

Note: raising concerns refers to “speaking up” or reporting by healthcare professionals for the benefit of patient safety and quality of care upon recognizing or becoming aware of any unprofessional, unethical or deficient actions from individuals within the healthcare setting. This may include clinical, performance or personal misconduct.

3.4 *How confident would you feel reporting an issue regarding attitude and conduct between clinical staff, for example, an argument?*

☐ Least confident ☐ Not confident ☐ Neutral ☐ Fairly confident ☐ Very confident

Rate your confidence in reporting this issue to each of the following:

a. Clinical supervisor
☐ Least confident ☐ Not confident ☐ Neutral ☐ Fairly confident ☐ Very confident

b. Dental nurse/ assistant
☐ Least confident ☐ Not confident ☐ Neutral ☐ Fairly confident ☐ Very confident

c. Academic mentor/ course coordinator
☐ Least confident ☐ Not confident ☐ Neutral ☐ Fairly confident ☐ Very confident

d. Fellow student
☐ Least confident ☐ Not confident ☐ Neutral ☐ Fairly confident ☐ Very confident

e. Clinical manager (Head of clinics)
☐ Least confident ☐ Not confident ☐ Neutral ☐ Fairly confident ☐ Very confident

3.5 *How confident would you feel reporting an issue regarding attitude and conduct of clinical staff toward a student, such as disrespect?*

☐ Least confident ☐ Not confident ☐ Neutral ☐ Fairly confident ☐ Very confident

Rate your confidence in reporting this issue to each of the following:

a. Clinical supervisor
☐ Least confident ☐ Not confident ☐ Neutral ☐ Fairly confident ☐ Very confident

b. Dental nurse/ assistant
☐ Least confident ☐ Not confident ☐ Neutral ☐ Fairly confident ☐ Very confident

c. Academic mentor/ course coordinator
☐ Least confident ☐ Not confident ☐ Neutral ☐ Fairly confident ☐ Very confident

d. Fellow student
☐ Least confident ☐ Not confident ☐ Neutral ☐ Fairly confident ☐ Very confident

e. Clinical manager (Head of clinics)
☐ Least confident ☐ Not confident ☐ Neutral ☐ Fairly confident ☐ Very confident

Rate your confidence in reporting this issue to each of the following:

a. Clinical supervisor
☐ Least confident ☐ Not confident ☐ Neutral ☐ Fairly confident ☐ Very confident

b. Dental nurse/ assistant
☐ Least confident ☐ Not confident ☐ Neutral ☐ Fairly confident ☐ Very confident

c. Academic mentor/ course coordinator
☐ Least confident ☐ Not confident ☐ Neutral ☐ Fairly confident ☐ Very confident

d. Fellow student
☐ Least confident ☐ Not confident ☐ Neutral ☐ Fairly confident ☐ Very confident

e. Clinical manager (Head of clinics)
☐ Least confident ☐ Not confident ☐ Neutral ☐ Fairly confident ☐ Very confident

Note: raising concerns refers to “speaking up” or reporting by healthcare professionals for the benefit of patient safety and quality of care upon recognizing or becoming aware of any unprofessional, unethical or deficient actions from individuals within the healthcare setting. This may include clinical, performance or personal misconduct.

Section 4: Barriers to raising concerns and suggestions for institutional support

4. 1 Have you experienced any barriers to raising concerns at your institution?
(Select one or more options)

☐ I do not want to cause trouble

☐ I do not know whom to talk to

☐ I feel it will be difficult to prove

☐ I feel unsupported

☐ I fear of creating conflict (to a member of staff or colleague)

☐ I am worried it will negatively affect my grades

☐ I fear I would not be listened to / ignored

☐ I fear that others will perceive my speaking up as negative

☐ I am worried I might be wrong because I don't have enough experience

☐ I do not feel it is worth speaking up

☐ Others (Please explain) ------ --

4. 2 Which of the following would encourage people to raise concerns?
(Select one or more options)

☐ Assure safety for students who raise concerns

☐ Clarify areas which must be reported

☐ Ensure that changes will be applied following a report

☐ Introduce a clear channel for raising concerns

☐ Identify a designated person to report such concerns

☐ Offer regular training and support

☐ Others (Please explain) --

End

Thank you for your participation

Note: raising concerns refers to "speaking up" or reporting by healthcare professionals for the benefit of patient safety and quality of care upon recognizing or becoming aware of any unprofessional, unethical or deficient actions from individuals within the healthcare setting. This may include clinical, performance or personal misconduct.
